# Disease-associated pathophysiologic structures in pediatric rheumatic diseases show characteristics of scale-free networks seen in physiologic systems: implications for pathogenesis and treatment

**DOI:** 10.1186/1755-8794-2-9

**Published:** 2009-02-23

**Authors:** Mark Barton Frank, Shirley Wang, Amita Aggarwal, Nicholas Knowlton, Kaiyu Jiang, Yanmin Chen, Ryan McKee, Brad Chaser, Timothy McGhee, Jeanette Osban, James N Jarvis

**Affiliations:** 1 Microarray Research Facility, Arthritis and Immunology Program, Oklahoma Medical Research Foundation, Oklahoma City, OK, USA; 2 Pediatric Rheumatology Section, University of Oklahoma College of Medicine, Oklahoma City, OK, USA; 3 Clinical Immunology, Department of Immunology, Sanjay Gandhi Postgraduate Institute of Medical Sciences, Lucknow, India; 4 The University of Alabama, Tuscaloosa, AL, USA

## Abstract

**Background:**

While standard reductionist approaches have provided some insights into specific gene polymorphisms and molecular pathways involved in disease pathogenesis, our understanding of complex traits such as atherosclerosis or type 2 diabetes remains incomplete. Gene expression profiling provides an unprecedented opportunity to understand complex human diseases by providing a global view of the multiple interactions across the genome that are likely to contribute to disease pathogenesis. Thus, the goal of gene expression profiling is not to generate lists of differentially expressed genes, but to identify the physiologic or pathogenic processes and structures represented in the expression profile.

**Methods:**

RNA was separately extracted from peripheral blood neutrophils and mononuclear leukocytes, labeled, and hybridized to genome level microarrays to generate expression profiles of children with polyarticular juvenile idiopathic arthritis, juvenile dermatomyositis relative to childhood controls. Statistically significantly differentially expressed genes were identified from samples of each disease relative to controls. Functional network analysis identified interactions between products of these differentially expressed genes.

**Results:**

*In silico *models of both diseases demonstrated similar features with properties of scale-free networks previously described in physiologic systems. These networks were observable in both cells of the innate immune system (neutrophils) and cells of the adaptive immune system (peripheral blood mononuclear cells).

**Conclusion:**

Genome-level transcriptional profiling from childhood onset rheumatic diseases suggested complex interactions in two arms of the immune system in both diseases. The disease associated networks showed scale-free network patterns similar to those reported in normal physiology. We postulate that these features have important implications for therapy as such networks are relatively resistant to perturbation.

## Background

Genome-based technologies provide us with an unprecedented capacity to understand complex biological systems and their relationship to health and disease. This is especially true for complex biological traits (e.g., atherosclerosis, hypertension), which have largely eluded our understanding using conventional, reductionist approaches. Indeed, even single-gene traits have demonstrated previously unsuspected levels of complexity when scrutinized through the lens of whole-genome technologies [1-3]

Chronic inflammatory diseases such as rheumatoid arthritis (RA) and juvenile dermatomyositis (JDM) are examples of human diseases whose etiologies and pathogenic mechanisms remain incompletely understood. Once thought to be purely "autoimmune" diseases triggered by a breakdown of the mechanisms that distinguish "self" from "non-self," it is becoming increasingly clear that these diseases involve complex interactions between the adaptive immune system (where these distinctions are made and immunologic memory is "stored") and the innate immune system (the parts of the immune system that do not require prior antigen exposure for optimal function) [4,5]. We therefore had begun to investigate these diseases from a systems biology approach, in which multiple relevant biological/pathological pathways can be queried simultaneously and their changes observed, defined, and modeled [6,7].

Until recently, there were no biomedical tools available to facilitate taking this approach. Advances in miniaturization and robotics have made this approach feasible, providing the opportunity to address critical questions of pediatric rheumatic disease pathogenesis, diagnosis, prognosis, and identification of targets of therapy in this "global" way. This understanding, in turn, is critical to our understanding the disease and our translation of that understanding into clinical practice. Of the available genome-wide technologies, gene expression microarrays are in the most mature phase of development, allow the most rigorous level of independent corroboration, and show the greatest promise for rapid translation into the clinical sphere [8].

One of the potential strengths of the currently available systems biology tools is the capacity to identify pathologic networks that underlie disease phenomena. Specifically, gene expression profiling has the capacity to do more than generate lists of differentially expressed genes; it provides an opportunity to observe gene regulation across the genome for patterns associated with health and disease. The current study was aimed at testing the feasibility of using gene expression profiling as a first step in understanding the structure of pathogenic networks in a family of illnesses collectively called *childhood onset rheumatic diseases*, particularly those diseases that are unique to childhood: juvenile idiopathic arthritis and juvenile dermatomyositis. Our findings have important implications to both our understanding of disease pathogenesis and to development of new therapies for these perplexing diseases.

## Methods

### Patient populations and control subjects

All human subject involvement in this study was reviewed and approved by the University of Oklahoma Health Science Center Institutional Review Board. As a proof-of-concept for the use of gene expression profiling and *in silico *modeling to identify pathologic and interconnecting structures, we studied two pediatric inflammatory diseases of diverse phenotype.

#### Juvenile idiopathic arthritis (JIA)

Children with this disease present with joint inflammation and synovial proliferation. Children in this study (12 females and 2 male; ages 3 – 13 years) were limited to those who fit criteria for the polyarticular, rheumatoid factor-negative subtype as defined by the International League Against Rheumatism [9]. All children had active disease as defined by consensus criteria developed by Wallace *et al *[10].

#### Juvenile dermatomyositis (JDM)

Children with juvenile dermatomyositis present with insidious onset of weakness and display a characteristic rash. Histopathology of affected muscle shows a distinct vasculitis and perivascular infiltrate composed primarily of lymphocytes and monocytes [11]. Children in this study (9 females and 8 males; ages 7 – 18 years) all fit criteria for diagnosis as established by Bohan and Peter [12] and had active disease at the time of study.

#### Pediatric controls

Children undergoing elective surgery for non-inflammatory conditions (e.g., removal of an orthopedic appliance, cosmetic surgery for pectus excavatum, etc; 9 females and 2 males, ages 7 – 18 years) were used as controls for this study. Screening questions excluded children with past histories of arthritis and/or muscle inflammation and children with first degree relatives with systemic lupus, rheumatoid arthritis (adult or childhood onset) or inflammatory muscle disease. Blood specimens from control children were handled in a manner identical to the handling of patient specimens.

### Specimens and processing

Following the execution of the informed consent process as approved by the University of Oklahoma Health Sciences Center (OUHSC) Institutional Review Board, 20 mL of whole blood was drawn into sterile sodium citrate tubes containing a cell density gradient (BD Biosciences Vacutainer^® ^CPT™ Cell Preparation Tube, # 362761, San Diego, CA, USA) and carried immediately to the Pediatric Rheumatology Research laboratories on the OUHSC campus. Granulocytes were immediately separated from mononuclear cells by density gradient centrifugation. Centrifugation was performed at room temperature. Mononuclear cells were removed from the density gradient interface and placed immediately in TRIZOL^® ^Reagent (Invitrogen, Carlsbad, CA, USA). Granulocytes and red cells layered in the bottom of the tube were separately collected. Red cells were removed from the granulocytes by hypotonic cell lysis as recommended by the manufacturer, and granulocytes were placed immediately in TRIZOL^® ^reagent for RNA purification. This method has been shown to provide the least amount of artifact in neutrophils prepared for microarray analysis [13,14].

### Microarray platform

Affymetrix human U133 Plus 2.0 GeneChip^® ^microarrays were used for all samples (Affymetrix, Inc., Santa Clara, CA). This platform contains oligonucleotides representing approximately 47,000 transcripts, including alternative splice variants of selected mRNAs.

### RNA purification, labeling, hybridization, and scanning

Total RNA extractions from Trizol^® ^reagent were carried out according to manufacturer's directions. RNA was further purified by passage through RNeasy mini-columns (QIAGEN, Valencia, CA) according to manufacturer's protocols for RNA clean-up. Final RNA preparations were suspended in RNase-free water. RNA was quantified spectrophotometrically. RNA integrity was assessed using capillary gel electrophoresis (Agilent 2100 Bioanalyzer; Agilent Technologies, Inc., Palo Alto, CA, USA) to determine the ratio of 28s:18s rRNA in each sample. A ratio greater than 1.0 was used to define samples of sufficient quality, and only samples above this limit were used for microarray studies. Four samples from neutrophils obtained from controls, 2 samples from PBMC obtained from controls, 1 sample from PBMC obtained from patients with JIA, and 1 sample from PBMC obtained from a patient with JDM were excluded from the study for this reason. No statistically significant differences in RNA quality were found within cell types between patients and controls. cDNA synthesis, hybridization and staining were performed as specified by Affymetrix (Santa Clara, CA). Briefly, 5 ug of total RNA was primed with T7-oligo-dT and reverse transcribed with SuperScript II, followed by production of double-stranded cDNA with *E coli *DNA polymerase. cRNA was transcribed *in vitro *from the T7 promoter using a biotinylated ribonucleotide analog and then fragmented to approximately 100 nt. cRNA was hybridized to Affymetrix GeneChips described above, and then washed and stained using an Affymetrix automated GeneChip^® ^450 fluidics station. Microarrays were scanned with an Affymetrix 3000 7G scanner.

### Statistical analysis

Data pre-processing was performed in the R/Bioconductor Package, "Affy". The raw Affymetrix perfect match probes were normalized by the RMA method combined with median-polish [15]. The marginal data distributions were adjusted through quantile normalization. The resulting normalized values were imported into BRB ArrayTools (Biometric Research Branch, National Cancer Institute) where they were then log transformed. Genes were filtered using the "Log Expression Variation Filter" to screen out genes that are not likely to be informative, based on the variance of each gene across the arrays. In this case, the filter was set to exclude genes that fell below the 50th percentile of gene variance. We identified genes that were differentially expressed between the two classes by using a multivariate permutation test [16,17]. We used the multivariate permutation test to provide a p value < 0.001. The test statistics used were random variance t-statistics for each gene [18]. Although t-statistics were used, the multivariate permutation test is non-parametric and does not require the assumption of Gaussian distributions. Data were exported to Excel (Microsoft, Redmond, WA) where averages of the classes were used to calculate expression ratios. Genes that simultaneously were differentially expressed (p < 0.001), a between class ratio of 1.8-fold or larger, and minimum normalized average intensity > 64 units in at least one group were retained for further analysis. Unsupervised hierarchical clustering of both samples and genes was performed in Spotfire (Sommerville, MA) after z-transformation of the data using Ward's minimum variance method [19]. Differences between cluster groups will be tested through a Chi-Square test. A p-value less than 0.05 was considered statistically significant.

### Reverse transcription – quantitative real-time PCR validation

Four differentially expressed genes in JIA patients relative to controls were tested using quantitative real-time PCR, namely prostrate acid phosphatase (ACPP), CUG triplet repeat RNA binding protein 2 (CUGBP2), coagulation factor XIII A1 polypeptide (F13A1), and cortactin (CTTN). The human glyceraldehyde 3-phosphate dehydrogenase (GAPDH) gene was used as an internal control. For JDM, myeloid cell leukemia sequence 1 (MCL1), proteasome 26S subunit, non-ATPase 7 (PSMD7), CD160, granzyme K (GZMK), and v-myb myeloblastosis viral oncogene homolog (avian)-like 1 (MYBL1) were tested. Total RNA was isolated as described above. Primers were designed with a 60°C melting temperature and a length of 9–40 nucleotides to produce PCR products with lengths between 50–150 bp using Applied Biosystems' Primer Express 2.0 software (Applied Biosystems Inc., Foster City, CA). First strand cDNA was generated from 1.8 ug of total RNA per sample using OmniScript Reverse Transcriptase according to manufacturer's directions (QIAGEN, Valencia, CA). cDNA was diluted 1:20 in water. PCR was run with 4 μl cDNA template in 20 μl reactions in duplicate on an ABI SDS 7000 using the ABI SYBR Green I Master Mix and gene specific primers at a concentration of 0.2 μM each. The temperature profile consisted of an initial 95°C step for 10 minutes, followed by 40 cycles of 95°C for 15 sec, 60°C for 1 min, and then a final melting curve analysis with a ramp from 60°C to 95°C over 20 min. Gene-specific amplification was confirmed by a single peak using the ABI Dissociation Curve software. Average Ct values for GAPDH (run in parallel reactions to the genes of interest) were used to normalize average Ct values of the gene of interest. Relative ΔCt was used to calculate fold-change values between groups.

### Pathway analysis

Pathways were generated by placing only statistically significantly differentially expressed genes with a minimum 1.8-fold difference between groups into Ingenuity Pathways Analysis (Ingenuity Systems^®^, Redwood City, CA). Each Affymetrix gene identifier was mapped to its corresponding gene object in the Ingenuity Knowledge Base. These "focus" genes were overlaid onto a global molecular network developed from information contained in the Ingenuity Knowledge Base. The software then algorithmically developed networks around these focus genes based "connectivity" derived from known interactions between products of these genes. No additions of molecules to these networks were made by authors. Network score values listed in figure legends are the -log_10 _(p-value) of finding this number of focus genes in a group of randomly selected genes the size of a given network.

## Results

### Gene expression differences in neutrophils and PBMC distinguish patients with juvenile onset rheumatic diseases from controls

Microarray data from isolated peripheral blood neutrophil RNA were obtained from 14 patients with active polyarticular JIA and from 13 control subjects. All data are available at NCBI's Gene Expression Omnibus (GEO Series Record GSE11083). Sixty genes were statistically significantly differentially expressed between these groups (Additional File 1). Nine of these genes were over-expressed in patients (1.8 – 3.0-fold different), and 51 were under-expressed in patients (1.8 – 2.5-fold different). Analysis of data from neutrophil samples from 14 patients with JDM and 13 controls revealed fifteen differentially expressed genes (Additional File 2), five that were under-expressed in patients (1.8 – 2.3 fold) and ten that were over-expressed in patients (1.8 – 2.4 fold different).

RNA isolated from PBMC from 15 patients with JIA, 13 patients with JDM, and 15 controls were used to obtain PBMC microarray datasets. The number of differentially expressed genes in PBMC was higher than in neutrophils for either disease relative to controls. Specifically, 128 genes differed in expression patterns between PBMC of JIA and controls (Additional File 3). 26 were under-expressed (1.8 – 2.6 fold different) and 102 were over-expressed (1.8 – 5.0 fold different) in patients. Twenty-six genes were statistically different between the JDM and control PBMC samples (Additional File 4). All of these were under-expressed in patients (1.8 – 6.9 fold different) with the exception of a 1.9-fold over-expression of mitochondrial tumor suppressor 1 (MTUS1) in patients.

No genes were found that were differentially expressed in both PBMC and neutrophils from JDM patients relative to controls. For JIA, two genes were differentially expressed in both PBMC and neutrophils relative to controls, namely phosphatase and tensin homolog (PTEN, Genbank accession AK021487, Affymetrix probe set id 233314_at) that was under-expressed 1.9-fold in JIA neutrophils and 2.6-fold in JIA PBMC, and ankyrin repeat domain 10 (Genbank accession AW299775, Affymetrix probe set id 235008_at) that was under-expressed 2.0-fold in JIA neutrophils and 2.6-fold in JIA PBMC. Therefore, the majority of differentially expressed genes identified here are unique to particular types of leukocytes.

We also found a small number of genes that were differentially expressed in both diseases relative to controls within a given type of cell. Products of such genes with similar expression patterns in both diseases may function in overlapping inflammatory or autoimmune aspects of these conditions. Two differentially expressed neutrophil genes were found in both diseases, namely the transcript encoding the CUG triplet repeat, RNA binding protein 2 (AA700631; 1565599_at) that was 2.2- and 2.4-fold over-expressed in JIA and JDM respectively; and the transcript encoding phosphatidylinositol binding clathrin assembly protein (AW293296; 239102_s_at) that was 2.3- and 1.8-fold under-expressed in JIA and JDM, respectively. For PBMC, three genes were differentially expressed in both diseases relative to controls, namely granzyme K (GZMK) that was 1.9-fold under-expressed in both diseases, homeodomain-only protein (HOP) under-expressed 1.8-fold in both diseases, and v-myb myeloblsatosis viral oncogene homolog (avian)-like 1 (MYBL1) under-expressed 1.9-fold in both diseases. One other gene (225239_at) whose product has not been functionally characterized was also differentially under-expressed in PBMC from both diseases relative to controls. Based on these findings, gene expression patterns of these diseases are considerably different form one another.

**Figure 1:**
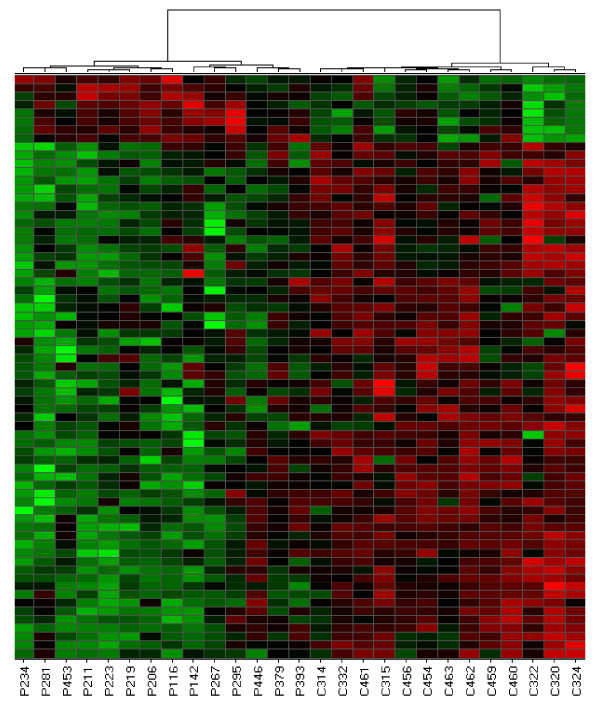
**Hierarchical cluster analysis derived from gene expression profiling in neutrophils of children with active polyarticular JIA and neutrophils from control children**. Clustering was performed using Ward's method which clustered on both the samples and the genes. Control samples are designated by the prefix "C", patient samples are designated "P". Each column represents patterns of genes from a separate sample, indicated by different numbers. Each row represents a separate gene. Red: genes that are over-expressed in disease; green: genes that are under-expressed in disease.

**Figure 2:**
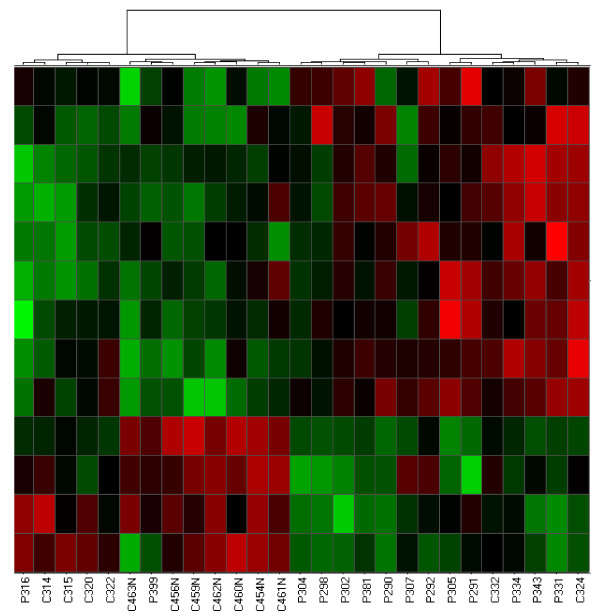
**Hierarchical cluster analysis derived from gene expression profiling of neutrophils from JDM and control samples**. Columns and rows are as described in Figure 1.

For both childhood-onset rheumatic diseases studied here, patients could be distinguished from control children by hierarchical cluster analysis of microarray data obtained from peripheral blood cells. Figure 1 shows a heat map of differentially expressed genes that were identified in neutrophils from patients with JIA and controls. All control samples clustered to the right side of the heat map while all patients clustered to the left. These results demonstrate that gene expression profiling of neutrophils distinguishes most children with JIA from a control population of children (X^2 ^= 23.14; p = 1.5 × 10^-6^). Hierarchical clustering of differentially expressed neutrophil genes also separated the majority of patients with JDM samples from controls. The cluster on the right side of Figure 2 represents data obtained from 12 patients and 2 controls, while the cluster on the left side contains data from 2 patients and 11 controls (X^2 ^= 10.69, p = 1.1 × 10^-3^).

**Figure 3:**
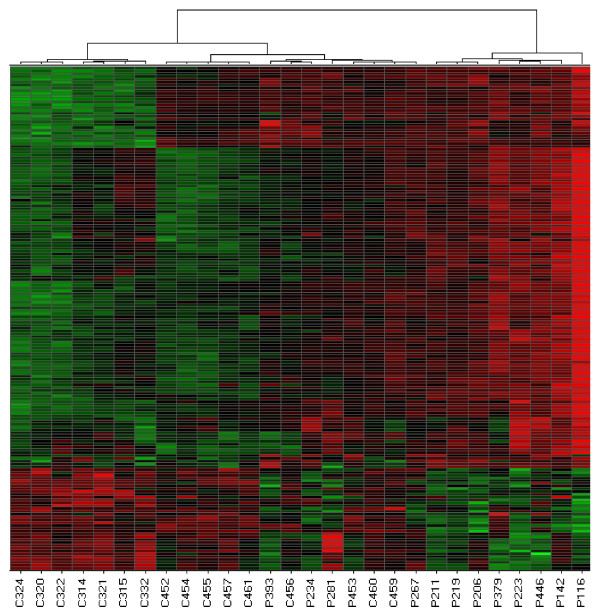
**Hierarchical cluster analysis derived from gene expression profiling of PBMC from JIA and control samples**. Columns and rows are as described in Figure 1.

**Figure 4:**
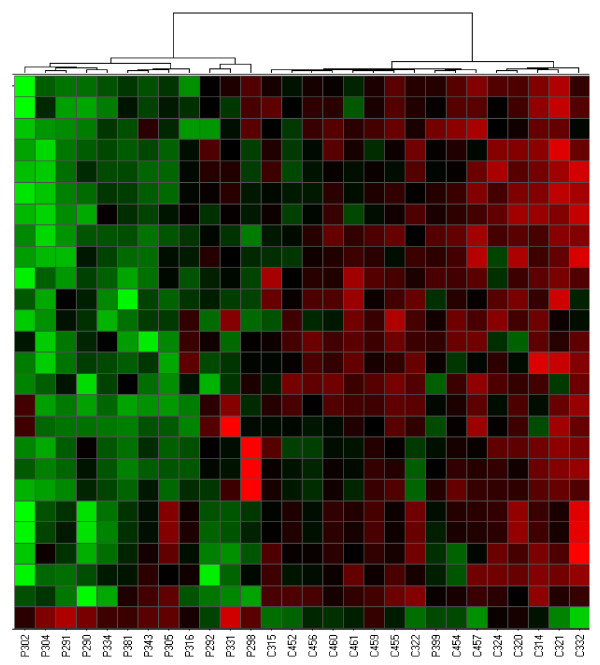
**Hierarchical cluster analysis derived from gene expression profiling of PBMC from JDM and control samples**. Columns and rows are as described in Figure 1.

Differentially expressed genes in PBMC were also good at discriminating either disease from controls using hierarchical clustering. For JIA, the left cluster in figure 3 contains samples from 15 controls and five patients, while the right cluster contains only samples from eight patients (X^2 ^= 10.08, p = 1.5 × 10^-3^). For JDM, the left cluster in Figure 4 contains 12 patient samples, while right cluster contains one patient and all control samples (X^2 ^= 20.61; p = 5.6 × 10^-6^). Patient 399 had active disease at the time the blood sample was donated for this study.

Nine differentially expressed genes selected from both cell types in both diseases were analyzed for differences using quantitative real-time PCR *(MCL1, PSMD7, CD160, GZMK, MYBL1, ACPP, CUGBP2, F13A1 and CTTN)*. In every case, those that were differentially over- or under-expressed on microarrays were similarly over- or under-expressed by quantitative PCR.

**Figure 5:**
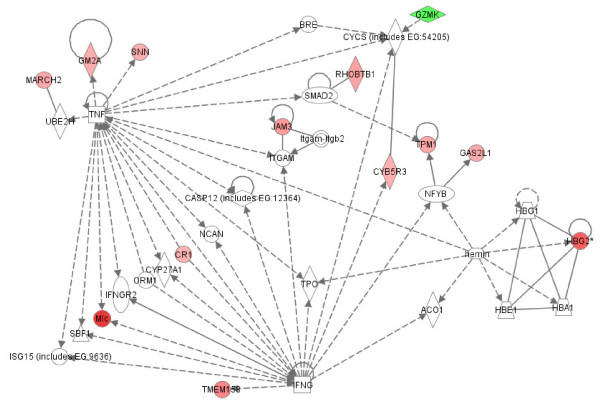
**Interactions between products of genes that were differentially expressed in JIA patient PBMC samples relative to controls**. Differentially expressed genes are designated by color (red: up-regulated in disease; green: down-regulated in disease). Increased intensity reflects greater fold-change between groups. Additional genes that were not differentially expressed between groups (not colored) with known interactions to differentially expressed genes were added by IPA software. Network score = 20.

**Figure 6:**
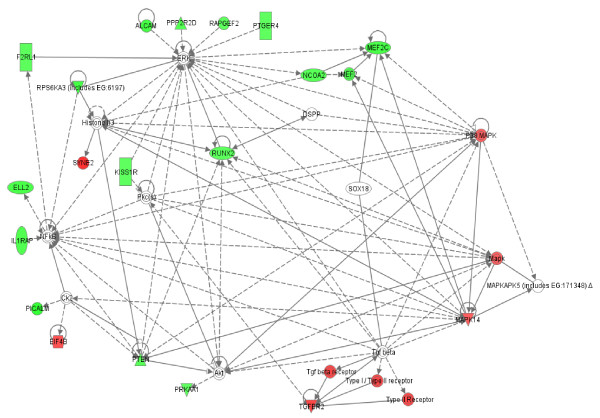
**Interactions between products of genes that were differentially expressed in JIA patient neutrophil samples relative to controls**. Network score = 17. Patterns are as described in the legend of Figure 5.

**Figure 7:**
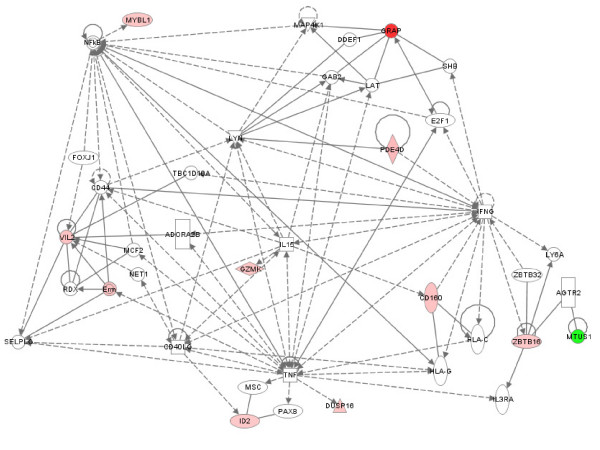
**Interactions between products of genes that were differentially expressed in JDM PBMC relative to control PBMC**. Network score = 23. Patterns are as described in the legend of Figure 5.

**Figure 8:**
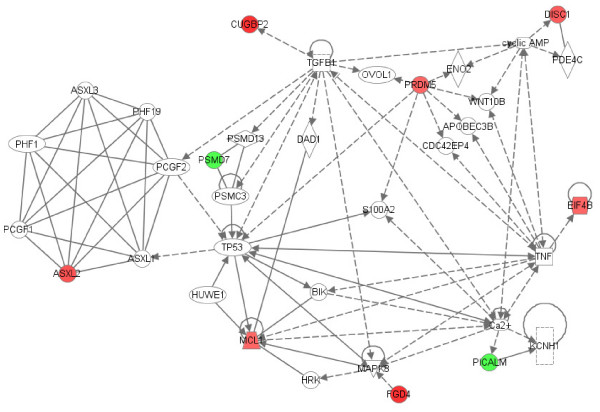
**Interactions between products of genes that were differentially expressed in JDM neutrophils relative to control neutrophils**. Network score = 25. Patterns are as described in the legend of Figure 5.

To better understand potential functional interactions between products of genes that were differentially expressed in these childhood-onset rheumatic diseases relative to childhood controls, differentially expressed genes were analyzed *in silico *using the Ingenuity Pathways Analysis (IPA) software (Ingenuity^® ^Systems, Inc., Redwood City, CA). For each disease and cell type, we observed multiple interconnected networks between gene products, a feature designated *modularity *that is characteristic of normal metabolic networks. [20]. Within networks, certain gene products had numerous known interactions with other products (high connectivity), while other products had relatively few interactions. Figures 5, 6, 7, 8 contain representative examples. Of the 128 genes that were differentially expressed in PBMC from patients with JIA relative to controls, seven networks were identified that each contained twelve or more differentially expressed genes. Figure 5 shows high connectivity for tumor necrosis factor (TNF) and interferon gamma (IFNG) and lower connectivity for most other genes (e.g., SMAD2). The score calculated by Ingenuity Pathway Analysis software for this network is 20, indicating the probability of finding this number of focus genes in a randomly selected group of genes of this network's size is <10^-20^. (Network scores for all networks shown are listed in the figure legends.) Figure 6 shows results derived from gene expression differences in neutrophils from JIA patients relative to controls. Of the 60 genes that were differentially expressed, IPA identified 4 networks that each contained at least 9 differentially expressed genes. Here, NFKb and the kinases ERK, P38MAPK and MAPK14 show high connectivity. These kinases have been linked to the generation of reactive oxygen species in rheumatoid arthritis synovium [21]. In network theory, nodes with high connectivity are defined as *hubs *[22]. These results demonstrate that hubs may be represented by both non-differentially expressed (e.g., Figure 5) and differentially expressed genes (e.g., Figure 6). These transcriptional hubs may be connected to nodes that represent differentially expressed (shown in color) and similarly expressed genes. All seven networks generated from the JIA PBMC data and all 4 networks generated from the JIA neutrophil data contain such hub and node structures.

Such networks are not restricted to disease-associated transcriptional changes in JIA. Gene expression profiles in PBMC obtained from comparing patients with JDM to controls revealed TNF, nuclear factor kappa B (NFkB) and interferon gamma (IFNG) hubs from similarly expressed genes and a VIL2 hub from one of the differentially expressed genes between these groups (Figure 7). The TNF hub was also observed in a JDM neutrophil-derived network (Figure 8). A type 1 interferon signature has already been reported in JDM muscle [23] and peripheral blood of both adults and children with inflammatory mypoathy [24]. Polymorphisms in the TNFA gene are known to confer disease risk and are associated with differences in clinical course and disease outcome in JDM.

The functional modeling of differentially expressed genes illustrated here has identified long-suspected pathologic hubs centering on TNFα and IFNγ, and, in addition, a previously unknown (but biologically sensible) hub for proteins such as VIL2. These results demonstrate that hub and node structures, also known as *scale-free networks *[25] that have been described for normal metabolic processes are also observed in both neutrophils and PBMC gene expression signatures of these diseases.

## Discussion

One important insight that has emerged from the genomic era of biology and medicine has been the development of deeper insights into the complexity of biological structures and processes. Prior to this era, biologists were able to work under the assumption that biological processes were essentially linear and that different components of inter-related metabolic systems functioned according to classical network theory as articulated by Erdos and Renyi [26]. This theory assumes that each pair of constituents (nodes or vertices) in a network is connected in a linear fashion to each other by edges. Furthermore, each node has generally the same number of links with the others, and that the number of links follows a Poisson distribution depending on the number of constituents in the system. However, computer modeling of biological systems has challenged that concept [22]. Evidence from genome sequencing and known biochemical functions of specific proteins suggests that specific nodes within biologic networks show high degrees of connectivity to other components ("hubs"), while most other nodes show low degrees of connectivity [27]. This high variability in degrees between nodes is a general feature of scale-free networks, particularly when these degrees follow a power law distribution [28]. Furthermore, groups of metabolic networks can be grouped into larger elements of related function [29].

This paper is the first to report in a systematic way that *pathologic *networks carry many of the same features as physiologic networks. *A priori*, there is no reason to assume that disease states would imitate normal cellular function in its display of scale-free properties with modularity. It is possible, for example, that disease states might result in perturbation of cell function(s) such that a loss of scaling and increased linearity of metabolic networks is seen. Implicit in these models is the notion that chronic inflammatory diseases like JIA and JDM represent not the *breakdown *of normal immune mechanisms (as is posited in autoimmune theories of pathogenesis), but their *adaptive functioning*. This is a profoundly different pathogenic model and underscores the potential of systems biology thinking to transform our understanding of illnesses.

The fact that such scale-free networks exist among disease states has interesting implications for the chronic inflammatory diseases studied here. One of the most important features of scale-free networks is their relative resistance to perturbation or attack when peripheral nodes are targeted [30]. Only alterations in the hubs results in significant alteration in the network. This fact has obvious therapeutic implications for these diseases. It should be clear that the most promising targets of therapy are going to be those specifically directed at pathologic hubs. Even if a gene shows strong differential expression between children with disease and control children, that gene is unlikely to be a promising therapeutic target if it is a more peripheral node. Proof-of-concept for this idea comes from our clinical experience with JIA. TNF inhibitors have emerged in the past 20 years as the most highly successful therapy for this disease [31], and we note that in our analysis, TNF is a prominent hub in a pathology-associated metabolic network in both neutrophils and PBMC (Figures 6, 7, 8). Based on this model, we would predict that targeting a gene such as JAM3 (Figure 5), although highly differentially expressed between children with JIA and controls, would be a less successful strategy.

These studies also have implications for our understanding of the pathogenesis of rheumatic diseases in children. In the diseases studied here, alterations in gene expression profiles relative to control subjects occurred in cells of both innate (neutrophils) and adaptive (PBMC) immunity. These findings suggest that complex interactions between innate and adaptive immunity occur in these diseases, as we have previously proposed [8,32]. Thus, it is perhaps an over-simplification to refer to these illnesses as "autoimmune" disorders, and, indeed, little in the expression profiles or the *in silico *models supports the hypothesis that these illnesses are driven primarily by a breakdown in the mechanisms through which "self" and "non-self" are recognized within the adaptive immune system. Rather, the metabolic structures may be better interpreted as an adaptation to an externally-applied force or forces (a hypothesis that does not exclude the possibility that one of those elements is an altered or inappropriately recognized self antigen).

There are some limitations on these data that must be considered. The most important limitation concerns the Ingenuity Pathway Analysis (IPA) program itself. The program builds its models by querying the known literature. This has several implications. First, the models reflect what is already known about interactions between specific genes. Unknown interactions could not be discovered through this analysis, and thus it is likely that there are highly relevant interactions that do not emerge in IPA. The next limitation is the fact that, since IPA queries only known associations and interactions, genes about which little or nothing is known about the function of their products cannot be identified as hubs using this method. Given these limitations, the models generated here must be considered preliminary and incomplete. However, despite these limitations, we believe that the models that emerge from this analysis better explain the complexity encountered in the clinic than the more limited, linear models generated from purely reductionist approaches.

## Conclusion

In conclusion, we demonstrate here that the pathology-associated metabolic networks in leukocytes of children with diverse inflammatory disorders show characteristics of *physiologic *cell networks. The scale-free nature of the hub-and-node structures of these networks provides new opportunities to identify targets of therapy and test models of disease pathogenesis. While the specific models reported here are likely to be altered as more is learned about the function of specific elements within the networks, the emergence of known points of pathogenic control within these networks (e.g., TNF) support the validity of the concepts reported here.

## Competing interests

The authors declare that they have no competing interests.

## Authors' contributions

MBF directed all aspects of microarray operations, performed data analysis and interpretation, and wrote the manuscript with JNJ; SW, AA, and TM assisted in patient characterization and microarray analyses on the JDM population; SW and AA also assisted in data analysis and interpretation; KJ and YC assisted cell separation and isolating RNA, and performed rtPCR studies; NK performed statistical analyses to identify differentially expressed genes; RM and BC assisted in identifying clinical phenotypes in the JIA population; JO performed RNA quality control, labeling, hybridizations and scanning; JNJ was the P.I. on this study and was responsible for the overall study design, data analysis and interpretation.

## Pre-publication history

The pre-publication history for this paper can be accessed here:



## Supplementary Material

Additional File 1 **Table 1.** Differentially expressed genes in jia v control neutrophils. Click here for file

Additional File 2 **Table 2.** Differentially expressed genes in jdm v control neutrophils. Click here for file

Additional File 3 **Table 3.** differentially expressed genes in jia v control pbmc. Click here for file

Additional File 4 **Table 4.** Differentially expressed genes in jdm v control pbmc. Click here for file
