# Supplementary material for: Disease-associated pathophysiologic structures in pediatric rheumatic diseases show characteristics of scale-free networks seen in physiologic systems: implications for pathogenesis and treatment
Source: BMC Med Genomics. 2009 Feb 23;2:9. doi: 10.1186/1755-8794-2-9 (PMC2649160; doi:10.1186/1755-8794-2-9)
Supplement: Additional File 1 — Table 1. Differentially expressed genes in jia v control neutrophils. [file 1755-8794-2-9-S1.doc]

Table 1: DIFFERENTIALLY EXPRESSED GENES IN JIA v CONTROL NEUTROPHILS

| **Gene Symbol** | **Gene Title** | **Probe** | **Control** | **JIA** | **Fold Change** | **p-value** |
| --- | --- | --- | --- | --- | --- | --- |
| ACPP | acid phosphatase, prostate | 204393_s_at | 420.53 | 215.68 | -1.95 | 5.10 |
| ALCAM | activated leukocyte cell adhesion molecule | 240655_at | 83.87 | 40.61 | -2.07 | 4.46 |
| ANKRD10 | ankyrin repeat domain 10 | 235008_at | 105.92 | 52.79 | -2.01 | 6.52 |
| ARID2 | AT rich interactive domain 2 (ARID, RFX-like) | 1553349_at | 202.09 | 114.40 | -1.77 | 4.91 |
| ATP2C1 | ATPase, Ca++ transporting, type 2C, member 1 | 230387_at | 195.57 | 106.01 | -1.84 | 4.52 |
| AZIN1 | antizyme inhibitor 1 | 240231_at | 174.86 | 86.53 | -2.02 | 4.04 |
| BBX | bobby sox homolog (Drosophila) | 1557240_a_at | 230.60 | 124.66 | -1.85 | 3.58 |
| CHD2 | chromodomain helicase DNA binding protein 2 | 228999_at | 228.20 | 127.33 | -1.79 | 3.71 |
| CNIH4 | cornichon homolog 4 (Drosophila) | 223993_s_at | 873.73 | 1533.10 | 1.75 | 3.41 |
| CUGBP2 | CUG triplet repeat, RNA binding protein 2 | 1565599_at | 145.62 | 325.45 | 2.23 | 3.92 |
| DNAJC7 | DnaJ (Hsp40) homolog, subfamily C, member 7 | 1556053_at | 177.63 | 95.52 | -1.86 | 4.31 |
| EFHC1 | EF-hand domain (C-terminal) containing 1 | 225656_at | 183.50 | 87.88 | -2.09 | 3.63 |
| EIF4B | eukaryotic translation initiation factor 4B | 219599_at | 68.51 | 134.70 | 1.97 | 3.34 |
| ELL2 | elongation factor, RNA polymerase II, 2 | 240038_at | 174.90 | 86.58 | -2.02 | 3.09 |
| F2RL1 | coagulation factor II (thrombin) receptor-like 1 | 213506_at | 1510.11 | 838.52 | -1.80 | 3.19 |
| FCHSD2 | FCH and double SH3 domains 2 | 242563_at | 83.59 | 37.38 | -2.24 | 5.43 |
| HOOK3 | hook homolog 3 (Drosophila) | 226395_at | 116.18 | 63.47 | -1.83 | 6.70 |
| IL1RAP | interleukin 1 receptor accessory protein | 210233_at | 197.70 | 101.14 | -1.95 | 3.82 |
| ITPR2 | inositol 1,4,5-triphosphate receptor, type 2 | 240458_at | 343.32 | 174.37 | -1.97 | 6.52 |
| KISS1R | KISS1 receptor | 242517_at | 79.43 | 44.88 | -1.77 | 3.61 |
| LOC283588 | hypothetical protein LOC283588 | 1557113_at | 110.08 | 57.52 | -1.91 | 3.95 |
| LOC728177 | hypothetical protein LOC728177 | 230543_at | 68.38 | 204.91 | 3.00 | 4.99 |
| MALAT1 | metastasis associated lung adenocarcinoma transcript 1 (non-protein coding) | 227510_x_at | 541.75 | 1025.52 | 1.89 | 3.80 |
| MAPK14 | mitogen-activated protein kinase 14 | 211087_x_at | 258.08 | 474.10 | 1.84 | 3.24 |
| MDM1 | Mdm4, transformed 3T3 cell double minute 1, p53 binding protein (mouse) | 213761_at | 118.70 | 58.51 | -2.03 | 3.61 |
| MEF2C | myocyte enhancer factor 2C | 236395_at | 90.37 | 44.45 | -2.03 | 3.78 |
| MGAT5 | mannosyl (alpha-1,6-)-glycoprotein beta-1,6-N-acetyl-glucosaminyltransferase | 215528_at | 165.13 | 87.15 | -1.89 | 5.52 |
| MLL5 | myeloid/lymphoid or mixed-lineage leukemia 5 | 1556306_at | 476.64 | 249.93 | -1.91 | 5.44 |
| NCOA2 | nuclear receptor coactivator 2 | 233834_at | 236.89 | 126.45 | -1.87 | 5.92 |
| NSMAF | neutral sphingomyelinase (N-SMase) activation associated factor | 232148_at | 173.08 | 96.84 | -1.79 | 3.31 |
| P15RS | cyclin-dependent kinase 2B-inhibitor-related protein | 231502_at | 80.35 | 42.67 | -1.88 | 5.00 |
| PICALM | phosphatidylinositol binding clathrin assembly protein | 239102_s_at | 701.45 | 311.27 | -2.25 | 5.12 |
| PIK3C2A | phosphoinositide-3-kinase, class 2, alpha polypeptide | 241905_at | 162.58 | 87.03 | -1.87 | 4.27 |
| PKP4 | plakophilin 4 | 236752_at | 285.21 | 115.36 | -2.47 | 3.62 |
| PPP2R2D | protein phosphatase 2, regulatory subunit B, delta isoform | 225066_at | 118.28 | 61.67 | -1.92 | 4.33 |
| PRKAA1 | protein kinase, AMP-activated, alpha 1 catalytic subunit | 214917_at | 71.37 | 40.39 | -1.77 | 6.70 |
| PS1TP4 | HBV preS1-transactivated protein 4 | 226381_at | 200.53 | 113.66 | -1.76 | 4.74 |
| PTEN | phosphatase and tensin homolog (mutated in multiple advanced cancers 1) | 233314_at | 818.90 | 438.28 | -1.87 | 5.02 |
| PTGER4 | prostaglandin E receptor 4 (subtype EP4) | 204897_at | 1786.32 | 996.74 | -1.79 | 5.10 |
| QKI | quaking homolog, KH domain RNA binding (mouse) | 212636_at | 883.76 | 491.84 | -1.80 | 3.78 |
| RAPGEF2 | Rap guanine nucleotide exchange factor (GEF) 2 | 234033_at | 314.15 | 177.82 | -1.77 | 3.89 |
| RAPGEF6 | Rap guanine nucleotide exchange factor (GEF) 6 | 239646_at | 123.67 | 49.59 | -2.49 | 7.00 |
| RPS6KA3 | ribosomal protein S6 kinase, 90kDa, polypeptide 3 | 241460_at | 315.06 | 152.06 | -2.07 | 7.00 |
| RUNX2 | runt-related transcription factor 2 | 232231_at | 1030.66 | 572.89 | -1.80 | 4.72 |
| SAPS3 | SAPS domain family, member 3 | 228105_at | 351.92 | 164.28 | -2.14 | 6.70 |
| SCLT1 | Sodium channel and clathrin linker 1 | 236487_at | 281.79 | 143.66 | -1.96 | 6.22 |
| SFRS15 | splicing factor, arginine/serine-rich 15 | 222310_at | 64.20 | 36.19 | -1.77 | 3.45 |
| SKAP2 | src kinase associated phosphoprotein 2 | 242598_at | 902.19 | 460.60 | -1.96 | 3.88 |
| SLC25A37 | solute carrier family 25, member 37 | 221920_s_at | 2917.72 | 1202.33 | -2.43 | 3.52 |
| SYNE2 | spectrin repeat containing, nuclear envelope 2 | 242774_at | 135.15 | 316.25 | 2.34 | 3.08 |
| TGFBR2 | transforming growth factor, beta receptor II (70/80kDa) | 236419_at | 42.85 | 90.53 | 2.11 | 3.29 |
| TMF1 | TATA element modulatory factor 1 | 236829_at | 183.24 | 99.84 | -1.84 | 7.00 |
| TRIP12 | Thyroid hormone receptor interactor 12 | 244659_at | 285.23 | 138.40 | -2.06 | 5.70 |
| UBE2H | ubiquitin-conjugating enzyme E2H (UBC8 homolog, yeast) | 229483_at | 304.30 | 140.50 | -2.17 | 7.00 |
| ZCCHC7 | zinc finger, CCHC domain containing 7 | 234032_at | 313.83 | 164.97 | -1.90 | 4.87 |
| --- | Transcribed locus | 215144_at | 109.31 | 211.43 | 1.93 | 3.40 |
| --- | --- | 231302_at | 383.67 | 218.65 | -1.75 | 4.62 |
| --- | CDNA clone IMAGE:3639730 | 1557539_at | 135.73 | 74.82 | -1.81 | 3.23 |
| --- | Transcribed locus | 229810_at | 80.52 | 41.15 | -1.96 | 3.44 |
| --- | Transcribed locus | 230416_at | 226.45 | 101.46 | -2.23 | 3.48 |

Gene symbols and titles are provided when annotations are available. Probe: Affymetrix probe set ID. Control and disease values are mean normalized signal intensities. Positive fold-change values are genes over expressed in disease, negative fold-change values are genes under-expressed in disease. P-values are –log(10) transformed. Genes identified with more than one independent probe are listed only once.
