# Supplementary material for: Disease-associated pathophysiologic structures in pediatric rheumatic diseases show characteristics of scale-free networks seen in physiologic systems: implications for pathogenesis and treatment
Source: BMC Med Genomics. 2009 Feb 23;2:9. doi: 10.1186/1755-8794-2-9 (PMC2649160; doi:10.1186/1755-8794-2-9)
Supplement: Additional File 2 — Table 2. Differentially expressed genes in jdm v control neutrophils. [file 1755-8794-2-9-S2.doc]

**Table 2: DIFFERENTIALLY EXPRESSED GENES IN JDM v CONTROL NEUTROPHILS**

| **Gene Symbol** | **Gene Title** | **Probe** | **Control** | **JDMS** | **Fold Change** | **p-value** |
| --- | --- | --- | --- | --- | --- | --- |
| ASXL2 | additional sex combs like 2 (Drosophila) | 1555266_a_at | 42.01 | 83.04 | 1.98 | 4.27 |
| FGD4 | FYVE, RhoGEF and PH domain containing 4 | 242445_at | 28.20 | 68.68 | 2.44 | 3.14 |
| KIAA0256 | KIAA0256 gene product | 212451_at | 188.79 | 81.23 | -2.32 | 3.16 |
| KIAA0430 | KIAA0430 | 1558697_a_at | 56.86 | 121.90 | 2.14 | 3.56 |
| MCL1 | myeloid cell leukemia sequence 1 (BCL2-related) | 200796_s_at | 848.09 | 1493.13 | 1.76 | 3.74 |
| PRDM5 | PR domain containing 5 | 220792_at | 52.40 | 93.50 | 1.78 | 3.10 |
| PRO1843 | hypothetical protein PRO1843 | 219599_at | 59.03 | 108.76 | 1.84 | 3.01 |
| PSMD7 | Proteasome (prosome, macropain) 26S subunit, non-ATPase, 7 (Mov34 homolog) | 238738_at | 97.69 | 55.43 | -1.76 | 3.71 |
| TTC26 | tetratricopeptide repeat domain 26 | 219758_at | 14.35 | 25.94 | 1.81 | 3.30 |
| --- | Clone 23712 mRNA sequence | 1565599_at | 121.46 | 286.89 | 2.36 | 3.29 |
| --- | --- | 233207_at | 56.10 | 109.11 | 1.95 | 4.85 |
| --- | --- | 1558588_at | 71.33 | 134.73 | 1.89 | 3.68 |
| --- | Transcribed locus | 239102_s_at | 608.90 | 342.48 | -1.78 | 4.67 |
| --- | Transcribed locus | 231111_at | 44.69 | 24.99 | -1.79 | 3.07 |
| --- | CDNA FLJ11750 fis, clone HEMBA1005568 | 232344_at | 66.45 | 37.09 | -1.79 | 8.10 |
