# Supplementary material for: Disease-associated pathophysiologic structures in pediatric rheumatic diseases show characteristics of scale-free networks seen in physiologic systems: implications for pathogenesis and treatment
Source: BMC Med Genomics. 2009 Feb 23;2:9. doi: 10.1186/1755-8794-2-9 (PMC2649160; doi:10.1186/1755-8794-2-9)
Supplement: Additional File 3 — Table 3. differentially expressed genes in jia v control pbmc. [file 1755-8794-2-9-S3.doc]

Table 3: DIFFERENTIALLY EXPRESSED GENES IN JIA v CONTROL PBMC

| **Gene Symbol** | **Gene Title** | **Probe** | **Control** | **JIA** | **Fold Change** | **p-value** |
| --- | --- | --- | --- | --- | --- | --- |
| ABCC4 | ATP-binding cassette, sub-family C (CFTR/MRP), member 4 | 1554918_a_at | 45.92 | 97.68 | 2.13 | 3.70 |
| ABLIM3 | actin binding LIM protein family, member 3 | 205730_s_at | 70.11 | 146.74 | 2.09 | 4.10 |
| ALAS2 | aminolevulinate, delta-, synthase 2 (sideroblastic/hypochromic anemia) | 211560_s_at | 111.20 | 422.26 | 3.80 | 3.49 |
| ALOX12 | arachidonate 12-lipoxygenase | 207206_s_at | 159.61 | 384.41 | 2.41 | 4.26 |
| ANKRD10 | ankyrin repeat domain 10 | 235008_at | 201.40 | 76.98 | -2.62 | 5.60 |
| ANKRD9 | ankyrin repeat domain 9 | 230972_at | 92.63 | 190.25 | 2.05 | 3.79 |
| AQP10 | aquaporin 10 | 1555338_s_at | 56.09 | 129.16 | 2.30 | 4.45 |
| ARHGAP6 | Rho GTPase activating protein 6 | 206167_s_at | 81.12 | 158.58 | 1.95 | 3.22 |
| ATP2A3 | ATPase, Ca++ transporting, ubiquitous | 207521_s_at | 93.23 | 226.18 | 2.43 | 4.27 |
| BCL2L1 | BCL2-like 1 | 215037_s_at | 91.67 | 202.99 | 2.21 | 5.03 |
| BSG | basigin (Ok blood group) | 208677_s_at | 268.52 | 559.40 | 2.08 | 4.77 |
| C12orf39 | chromosome 12 open reading frame 39 | 229778_at | 53.89 | 134.92 | 2.50 | 3.08 |
| C19orf22 | chromosome 19 open reading frame 22 | 221764_at | 612.00 | 1167.86 | 1.91 | 5.25 |
| C1orf198 | chromosome 1 open reading frame 198 | 223063_at | 136.04 | 243.59 | 1.79 | 4.92 |
| C5orf4 | chromosome 5 open reading frame 4 | 220751_s_at | 106.11 | 246.96 | 2.33 | 4.57 |
| C6orf25 | chromosome 6 open reading frame 25 | 221342_at | 68.85 | 225.96 | 3.28 | 6.70 |
| CALD1 | caldesmon 1 | 212077_at | 47.87 | 130.68 | 2.73 | 3.10 |
| CCAR1 | cell division cycle and apoptosis regulator 1 | 239014_at | 319.85 | 159.18 | -2.01 | 7.00 |
| CDC14A | CDC14 cell division cycle 14 homolog A (S. cerevisiae) | 210742_at | 312.84 | 178.72 | -1.75 | 4.17 |
| CLU | clusterin | 208792_s_at | 729.73 | 1839.32 | 2.52 | 4.90 |
| CMTM5 | CKLF-like MARVEL transmembrane domain containing 5 | 230942_at | 168.00 | 368.08 | 2.19 | 4.44 |
| CPNE5 | copine V | 227189_at | 167.24 | 324.52 | 1.94 | 4.14 |
| CR1 | complement component (3b/4b) receptor 1 (Knops blood group) | 206244_at | 80.94 | 151.28 | 1.87 | 5.60 |
| CTDSPL | CTD (carboxy-terminal domain, RNA polymerase II, polypeptide A) small phosphatase-like | 201904_s_at | 97.84 | 187.44 | 1.92 | 3.68 |
| CTSA | cathepsin A | 200661_at | 1019.81 | 1921.23 | 1.88 | 4.89 |
| CTTN | cortactin | 214073_at | 63.66 | 153.44 | 2.41 | 3.94 |
| CUGBP2 | CUG triplet repeat, RNA binding protein 2 | 1556323_at | 314.59 | 177.53 | -1.77 | 4.82 |
| CYB5R3 | cytochrome b5 reductase 3 | 1554574_a_at | 229.91 | 440.19 | 1.91 | 5.82 |
| DAPK3 | death-associated protein kinase 3 | 203890_s_at | 49.94 | 90.07 | 1.80 | 4.85 |
| DSC2 | desmocollin 2 | 226817_at | 60.70 | 118.57 | 1.95 | 3.79 |
| DUSP16 | dual specificity phosphatase 16 | 1558739_at | 112.78 | 49.04 | -2.30 | 4.50 |
| EGF | epidermal growth factor (beta-urogastrone) | 206254_at | 44.07 | 86.51 | 1.96 | 3.72 |
| EPB49 | erythrocyte membrane protein band 4.9 | 204505_s_at | 117.76 | 254.70 | 2.16 | 4.88 |
| ESAM | endothelial cell adhesion molecule | 225369_at | 67.06 | 117.68 | 1.75 | 3.55 |
| F13A1 | coagulation factor XIII, A1 polypeptide | 203305_at | 1182.46 | 2433.81 | 2.06 | 4.51 |
| FAM118A | family with sequence similarity 118, member A | 219629_at | 64.09 | 170.72 | 2.66 | 4.62 |
| FBXL3 | F-box and leucine-rich repeat protein 3 | 242829_x_at | 396.63 | 223.11 | -1.78 | 3.42 |
| FHL1 | four and a half LIM domains 1 | 210299_s_at | 119.59 | 236.56 | 1.98 | 4.45 |
| FLJ13197 | hypothetical FLJ13197 | 219871_at | 117.17 | 66.25 | -1.77 | 4.51 |
| FLNA | filamin A, alpha (actin binding protein 280) | 213746_s_at | 676.75 | 1273.43 | 1.88 | 3.66 |
| FNBP1 | formin binding protein 1 | 230389_at | 639.53 | 346.80 | -1.84 | 4.12 |
| GAS2L1 | growth arrest-specific 2 like 1 | 209729_at | 118.94 | 241.94 | 2.03 | 4.99 |
| GFI1B | growth factor independent 1B (potential regulator of CDKN1A, translocated in CML) | 208501_at | 63.23 | 138.13 | 2.18 | 4.48 |
| GM2A | GM2 ganglioside activator | 33646_g_at | 94.16 | 188.19 | 2.00 | 4.09 |
| GMPR | guanosine monophosphate reductase | 204187_at | 80.14 | 197.84 | 2.47 | 5.46 |
| SEPT5 | septin 5 | 206655_s_at | 304.56 | 992.63 | 3.26 | 5.80 |
| GP9 | glycoprotein IX (platelet) | 206883_x_at | 133.21 | 269.87 | 2.03 | 4.30 |
| GSTM4 | glutathione S-transferase M4 | 204149_s_at | 41.99 | 78.42 | 1.87 | 4.13 |
| GTPBP2 | GTP binding protein 2 | 223789_s_at | 56.21 | 105.44 | 1.88 | 5.72 |
| GZMK | granzyme K (granzyme 3; tryptase II) | 206666_at | 1314.42 | 692.86 | -1.90 | 3.08 |
| HBG1 | hemoglobin, gamma A | 204848_x_at | 520.72 | 1977.20 | 3.80 | 3.39 |
| HBG2 | hemoglobin, gamma G | 204419_x_at | 487.54 | 1895.41 | 3.89 | 3.23 |
| HBQ1 | hemoglobin, theta 1 | 220807_at | 44.86 | 93.43 | 2.08 | 3.52 |
| HIST2H2BE | histone cluster 2, H2be | 202708_s_at | 310.41 | 605.07 | 1.95 | 3.02 |
| HOP | homeodomain-only protein | 211597_s_at | 910.51 | 513.03 | -1.77 | 5.18 |
| HSPC159 | galectin-related protein | 219998_at | 42.92 | 101.98 | 2.38 | 4.56 |
| ID2 | inhibitor of DNA binding 2, dominant negative helix-loop-helix protein | 213931_at | 659.92 | 347.40 | -1.90 | 3.83 |
| ITGA2B | integrin, alpha 2b (platelet glycoprotein IIb of IIb/IIIa complex, antigen CD41) | 206494_s_at | 252.78 | 1093.81 | 4.33 | 6.52 |
| ITGB3 | integrin, beta 3 (platelet glycoprotein IIIa, antigen CD61) | 204625_s_at | 67.91 | 260.47 | 3.84 | 6.40 |
| ITGB5 | integrin, beta 5 | 201125_s_at | 174.90 | 329.06 | 1.88 | 3.06 |
| JAK3 | Janus kinase 3 (a protein tyrosine kinase, leukocyte) | 211108_s_at | 31.17 | 64.10 | 2.06 | 5.11 |
| JAM3 | junctional adhesion molecule 3 | 231721_at | 26.81 | 66.03 | 2.46 | 3.31 |
| KLRB1 | killer cell lectin-like receptor subfamily B, member 1 | 214470_at | 3052.13 | 1666.50 | -1.83 | 3.29 |
| KLRC3 | killer cell lectin-like receptor subfamily C, member 3 | 207723_s_at | 212.97 | 107.97 | -1.97 | 3.01 |
| LEPR | leptin receptor | 209894_at | 57.97 | 106.31 | 1.83 | 3.29 |
| LRRN3 | leucine rich repeat neuronal 3 | 209840_s_at | 725.13 | 367.39 | -1.97 | 4.16 |
| LTBP1 | latent transforming growth factor beta binding protein 1 | 202729_s_at | 105.85 | 211.16 | 1.99 | 3.84 |
| MARCH2 | membrane-associated ring finger (C3HC4) 2 | 210075_at | 261.30 | 541.71 | 2.07 | 4.40 |
| MFAP3L | microfibrillar-associated protein 3-like | 210843_s_at | 36.67 | 71.35 | 1.95 | 3.60 |
| MGLL | monoglyceride lipase | 211026_s_at | 126.19 | 271.21 | 2.15 | 3.95 |
| MPL | myeloproliferative leukemia virus oncogene | 207550_at | 92.12 | 201.05 | 2.18 | 4.14 |
| MYBL1 | v-myb myeloblastosis viral oncogene homolog (avian)-like 1 | 213906_at | 944.84 | 499.86 | -1.89 | 3.41 |
| MYL9 | myosin, light chain 9, regulatory | 201058_s_at | 121.57 | 610.58 | 5.02 | 6.70 |
| NFE2 | nuclear factor (erythroid-derived 2), 45kDa | 209930_s_at | 257.78 | 454.22 | 1.76 | 3.26 |
| NRGN | neurogranin (protein kinase C substrate, RC3) | 204081_at | 1327.34 | 3344.71 | 2.52 | 4.42 |
| NUCB1 | nucleobindin 1 | 200646_s_at | 178.88 | 316.00 | 1.77 | 5.19 |
| PARVB | parvin, beta | 204629_at | 125.11 | 319.38 | 2.55 | 4.68 |
| PDCL3 | phosducin-like 3 | 243049_at | 160.78 | 89.98 | -1.79 | 4.55 |
| PDLIM1 | PDZ and LIM domain 1 (elfin) | 208690_s_at | 695.90 | 1251.73 | 1.80 | 4.36 |
| PRKAR2B | protein kinase, cAMP-dependent, regulatory, type II, beta | 203680_at | 1158.07 | 2079.76 | 1.80 | 3.15 |
| PROS1 | protein S (alpha) | 207808_s_at | 64.77 | 146.70 | 2.26 | 4.21 |
| PRSS2 | protease, serine, 2 (trypsin 2) | 241133_at | 269.29 | 686.17 | 2.55 | 4.27 |
| PRSS23 | protease, serine, 23 | 226279_at | 104.02 | 54.76 | -1.90 | 3.01 |
| PRUNE | prune homolog (Drosophila) | 232490_s_at | 69.61 | 132.06 | 1.90 | 5.62 |
| PTCH1 | patched homolog 1 (Drosophila) | 209815_at | 147.75 | 79.81 | -1.85 | 4.98 |
| PTEN | phosphatase and tensin homolog (mutated in multiple advanced cancers 1) | 233314_at | 132.53 | 50.84 | -2.61 | 4.55 |
| PTGS1 | prostaglandin-endoperoxide synthase 1 (prostaglandin G/H synthase and cyclooxygenase) | 215813_s_at | 260.36 | 551.61 | 2.12 | 4.36 |
| PTPRJ | protein tyrosine phosphatase, receptor type, J | 210173_at | 42.23 | 77.14 | 1.83 | 6.52 |
| RHOBTB1 | Rho-related BTB domain containing 1 | 212651_at | 40.82 | 91.56 | 2.24 | 3.27 |
| RUFY1 | RUN and FYVE domain containing 1 | 233380_s_at | 391.50 | 761.43 | 1.94 | 3.56 |
| SAMHD1 | SAM domain and HD domain 1 | 1559882_at | 389.24 | 200.04 | -1.95 | 4.42 |
| SELP | selectin P (granule membrane protein 140kDa, antigen CD62) | 206049_at | 144.15 | 274.59 | 1.90 | 3.40 |
| SH3BGRL2 | SH3 domain binding glutamic acid-rich protein like 2 | 225354_s_at | 283.32 | 624.23 | 2.20 | 3.35 |
| SIGLEC5 | sialic acid binding Ig-like lectin 5 | 220000_at | 103.34 | 182.08 | 1.76 | 3.48 |
| SLC16A3 | solute carrier family 16, member 3 (monocarboxylic acid transporter 4) | 202855_s_at | 108.94 | 192.90 | 1.77 | 3.23 |
| SLC25A37 | solute carrier family 25, member 37 | 222528_s_at | 274.68 | 603.86 | 2.20 | 3.63 |
| SLC25A39 | solute carrier family 25, member 39 | 223649_s_at | 221.96 | 411.01 | 1.85 | 4.50 |
| SLC35E2 | solute carrier family 35, member E2 | 215169_at | 69.90 | 39.42 | -1.77 | 3.49 |
| SMOX | spermine oxidase | 1555680_a_at | 54.20 | 100.58 | 1.86 | 5.33 |
| SNCA | synuclein, alpha | 204466_s_at | 582.48 | 1367.57 | 2.35 | 3.59 |
| SNN | Stannin | 218033_s_at | 100.72 | 191.79 | 1.90 | 4.86 |
| SPARC | secreted protein, acidic, cysteine-rich (osteonectin) | 212667_at | 35.29 | 76.25 | 2.16 | 3.30 |
| SPI1 | spleen focus forming virus (SFFV) proviral integration oncogene spi1 | 205312_at | 268.99 | 473.43 | 1.76 | 3.06 |
| STRN4 | striatin, calmodulin binding protein 4 | 217903_at | 69.21 | 122.07 | 1.76 | 3.81 |
| STXBP2 | syntaxin binding protein 2 | 209367_at | 341.80 | 601.28 | 1.76 | 3.59 |
| SYTL3 | synaptotagmin-like 3 | 1562255_at | 99.52 | 46.43 | -2.14 | 5.08 |
| TAL1 | T-cell acute lymphocytic leukemia 1 | 206283_s_at | 171.96 | 302.67 | 1.76 | 3.18 |
| TGFB1I1 | transforming growth factor beta 1 induced transcript 1 | 209651_at | 25.54 | 84.45 | 3.31 | 5.55 |
| THBS1 | thrombospondin 1 | 201108_s_at | 243.30 | 558.12 | 2.29 | 4.18 |
| TMEM142B | transmembrane protein 142B | 218812_s_at | 61.25 | 112.39 | 1.84 | 5.07 |
| TMEM158 | transmembrane protein 158 | 213338_at | 69.05 | 202.10 | 2.93 | 5.17 |
| TPM1 | tropomyosin 1 (alpha) | 210987_x_at | 179.41 | 383.45 | 2.14 | 4.36 |
| TPM4 | tropomyosin 4 | 209344_at | 187.29 | 446.71 | 2.39 | 5.74 |
| TRAPPC1 | trafficking protein particle complex 1 | 225294_s_at | 773.96 | 1376.61 | 1.78 | 5.80 |
| TREML1 | triggering receptor expressed on myeloid cells-like 1 | 1555659_a_at | 330.85 | 801.21 | 2.42 | 4.38 |
| TRIM58 | tripartite motif-containing 58 | 215047_at | 183.05 | 461.89 | 2.52 | 4.68 |
| TTC7B | tetratricopeptide repeat domain 7B | 226152_at | 38.04 | 92.23 | 2.42 | 4.38 |
| TUBB1 | tubulin, beta 1 | 208601_s_at | 730.84 | 1617.72 | 2.21 | 5.11 |
| WASF3 | WAS protein family, member 3 | 204042_at | 35.57 | 89.79 | 2.52 | 3.51 |
| WBP2 | WW domain binding protein 2 | 209117_at | 264.16 | 462.28 | 1.75 | 4.28 |
| WDR1 | WD repeat domain 1 | 210935_s_at | 295.49 | 540.45 | 1.83 | 5.19 |
| XK | X-linked Kx blood group (McLeod syndrome) | 206698_at | 56.09 | 135.11 | 2.41 | 4.74 |
| YES1 | v-yes-1 Yamaguchi sarcoma viral oncogene homolog 1 | 222180_at | 68.98 | 33.22 | -2.08 | 4.50 |
| YIF1B | Yip1 interacting factor homolog B (S. cerevisiae) | 1554553_s_at | 187.89 | 340.87 | 1.81 | 4.45 |
| YWHAE | tyrosine 3-monooxygenase/tryptophan 5-monooxygenase activation protein, epsilon polypeptide | 210317_s_at | 193.29 | 339.48 | 1.76 | 3.97 |
| --- | Homo sapiens, clone IMAGE:4723617, mRNA | 1559916_a_at | 94.39 | 52.73 | -1.79 | 3.31 |
| --- | Transcribed locus | 230064_at | 76.57 | 42.56 | -1.80 | 3.52 |
| --- | CDNA FLJ26120 fis, clone SYN00419 | 225239_at | 1018.08 | 499.76 | -2.04 | 4.00 |
