# Supplementary material for: Disease-associated pathophysiologic structures in pediatric rheumatic diseases show characteristics of scale-free networks seen in physiologic systems: implications for pathogenesis and treatment
Source: BMC Med Genomics. 2009 Feb 23;2:9. doi: 10.1186/1755-8794-2-9 (PMC2649160; doi:10.1186/1755-8794-2-9)
Supplement: Additional File 4 — Table 4. Differentially expressed genes in jdm v control pbmc. [file 1755-8794-2-9-S4.doc]

**Table 4: DIFFERENTIALLY EXPRESSED GENES IN JDM v CONTROL PBMC**

| **Gene Symbol** | **Gene Title** | **Probe** | **Control** | **JDMS** | **Fold Change** | **p-value** |
| --- | --- | --- | --- | --- | --- | --- |
| CD160 | CD160 molecule | 207840_at | 282.5 | 141.1 | -2.0 | 3.75 |
| GZMH | granzyme H (cathepsin G-like 2, protein h-CCPX) | 210321_at | 1026.2 | 548.8 | -1.9 | 3.14 |
| GZMK | granzyme K (granzyme 3; tryptase II) | 206666_at | 1045.4 | 556.4 | -1.9 | 3.32 |
| HOP | homeodomain-only protein | 211597_s_at | 744.6 | 409.1 | -1.8 | 3.59 |
| ID2 | inhibitor of DNA binding 2, dominant negative helix-loop-helix protein /// inhibitor of DNA binding 2B, dominant negative helix-loop-helix protein | 213931_at | 509.9 | 282.7 | -1.8 | 5.49 |
| LOC144571 | hypothetical protein LOC144571 | 1564139_at | 285.3 | 107.7 | -2.6 | 3.67 |
| MIAT | myocardial infarction associated transcript (non-protein coding) | 228658_at | 190.6 | 103.0 | -1.8 | 6.24 |
| MTUS1 | mitochondrial tumor suppressor 1 | 212096_s_at | 44.0 | 84.3 | 1.9 | 4.23 |
| MYBL1 | v-myb myeloblastosis viral oncogene homolog (avian)-like 1 | 213906_at | 771.7 | 396.8 | -1.9 | 3.40 |
| TRGC2 / TRGV9 | T cell receptor gamma constant 2 /// T cell receptor gamma variable 9 | 216920_s_at | 1610.3 | 881.7 | -1.8 | 3.48 |
| TGFBR3 | transforming growth factor, beta receptor III | 204731_at | 275.4 | 108.5 | -2.5 | 6.22 |
| TRGV9 | T cell receptor gamma variable 9 | 209813_x_at | 1076.5 | 604.9 | -1.8 | 3.25 |
| ZBTB16 | zinc finger and BTB domain containing 16 | 205883_at | 110.9 | 60.6 | -1.8 | 4.12 |
| --- | --- | 226625_at | 478.4 | 268.4 | -1.8 | 3.92 |
| --- | Transcribed locus | 227762_at | 173.9 | 96.5 | -1.8 | 3.44 |
| --- | --- | 240652_at | 124.9 | 68.9 | -1.8 | 3.61 |
| --- | Transcribed locus | 243810_at | 145.0 | 79.4 | -1.8 | 4.34 |
| --- | CDNA FLJ37336 fis, clone BRAMY2020412 | 1558739_at | 85.9 | 46.0 | -1.9 | 3.14 |
| --- | CDNA FLJ26120 fis, clone SYN00419 | 225239_at | 784.5 | 417.5 | -1.9 | 3.91 |
| --- | Transcribed locus | 228854_at | 247.8 | 129.3 | -1.9 | 3.24 |
| --- | --- | 240188_at | 155.6 | 78.5 | -2.0 | 3.74 |
| --- | UG0651E06 | 1564248_at | 114.0 | 57.5 | -2.0 | 6.18 |
| --- | Transcribed locus | 214349_at | 132.5 | 60.0 | -2.2 | 3.13 |
| --- | Transcribed locus | 236610_at | 186.0 | 80.5 | -2.3 | 3.85 |
| --- | CDNA: FLJ20931 fis, clone ADSE01282 | 216050_at | 76.4 | 25.5 | -3.0 | 3.29 |
| --- | Transcribed locus | 235102_x_at | 230.6 | 33.6 | -6.9 | 4.07 |
